# Ribbon Crystals

**DOI:** 10.1371/journal.pone.0074932

**Published:** 2013-10-03

**Authors:** Jakob Bohr, Steen Markvorsen

**Affiliations:** 1 Department of Micro and Nanotechnology, Technical University of Denmark, Kongens Lyngby, Denmark; 2 Department of Applied Mathematics and Computer Science, Technical University of Denmark, Kongens Lyngby, Denmark; King's College London, United Kingdom

## Abstract

A repetitive crystal-like pattern is spontaneously formed upon the twisting of straight ribbons. The pattern is akin to a tessellation with isosceles triangles, and it can easily be demonstrated with ribbons cut from an overhead transparency. We give a general description of developable ribbons using a ruled procedure where ribbons are uniquely described by two generating functions. This construction defines a differentiable frame, the ribbon frame, which does not have singular points, whereby we avoid the shortcomings of the Frenet–Serret frame. The observed spontaneous pattern is modeled using planar triangles and cylindrical arcs, and the ribbon structure is shown to arise from a maximization of the end-to-end length of the ribbon, i.e. from an optimal use of ribbon length. The phenomenon is discussed in the perspectives of incompatible intrinsic geometries and of the emergence of long-range order.

## Introduction

Recently, Korte, Starostin, and van der Heijden reported on the fascinating occurrence of triangular buckling patterns in ribbons [1]. Following the method used in the study of the equilibrium shape of the Möbius strip [2], they elegantly built an analysis of the pattern on the triangular region of the Möbius strip [2] and used it to form a repetitive structure of triangular regions for the twisted ribbons [1]. The Möbius strip was modeled as a developable surface, and at a critical ratio of length to width it collapses into the expected triple covered equilateral triangle. At larger lengths it resembles the conical dislocations known from crumpling phenomena [2]–[4].

Here, we study the triangular tessellations of ribbons from a geometrical point of view and show that they are formed in the limit of an optimal (minimal) use of ribbon length. The spontaneous pattern partitions the ribbon, similarly to a tessellation, thereby transforming the continuous translational symmetry of a straight ribbon into a discrete symmetry where repeating ribbon segments are mapped onto each other via a screw translation. Further, we consider the properties of frames that can be associated with ribbons, and suggest the use of one which leads to a natural generation of all ribbons, henceforth denoted the ribbon frame.

The occurrence of long-range order is playing a significant role in many physical phenomena and structural phase transitions, e.g. in solidification into crystalline phases. In biology ordered and disordered tessellations appear ubiquitously. In mathematics there are numerous open questions in discrete geometry regarding tilings with concurrent elements [5]. In three dimensions new families of polyhedral tilings have recently been described [6], [7], and materials with interesting properties have been constructed with folded textured sheets [8], [9]. DNA ribbons have been formed from multiple molecules placed next to each other facilitating DNA origami and the formation of Möbius strips [10]. The differential geometry of ribbons have been considered in connection with statistical models for polymers [11]–[13]. An interesting recent application of ribbons is the reconstruction of two-dimensional surfaces from ribbon curves [14].

## Results and Discussion

**Figure 1:**
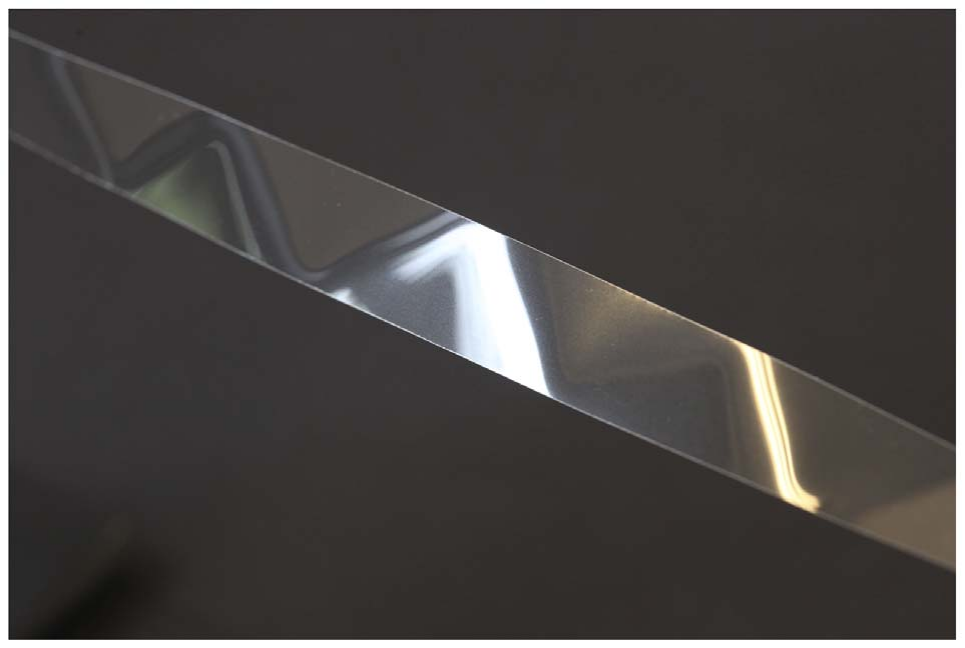
Triangular tessellation. Section of the triangular tessellation observed on a slightly twisted 18/3 turn over a length of 287 mm. Material: Xerox Premium Transparencies, Part no. 003R98202.

**Figure 2:**
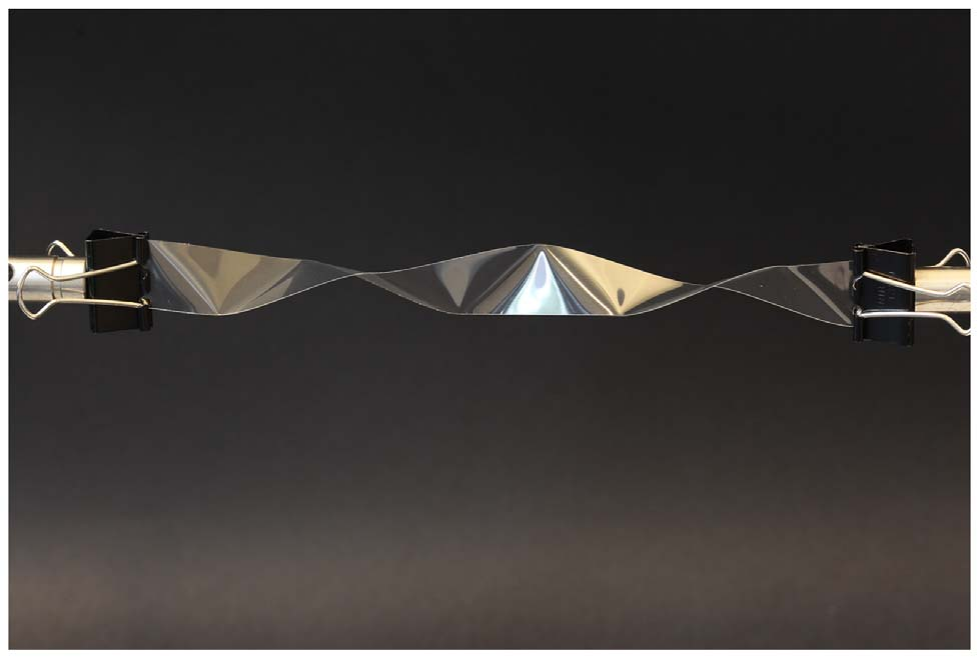
Ribbon with paper clips. Ribbon twisted about one full rotation over a length of 200

Figure 1 shows the spontaneous partitioning of a straight ribbon into repeating triangular-like structures upon a relatively small twisting of the ribbon. The ribbon, 18 mm wide, was cut from a commercially available size A4 overhead transparency (Xerox Premium Transparencies, Part no. 003R98202). Two paper grip clips mounted on standard laboratory stands are used to hold the ends of the ribbon, to control their relative rotation to each other, and to adjust the distance. With care the experiment can also be performed holding the ribbon just by hand using e.g. an edge of a table to fix one end of the ribbon. Figure 2 shows a more twisted ribbon where the triangles are also present. Visible are the two paper grip clips used to hold the ribbon in place. Also visible on the figures are what appear to be narrow strips of bent ribbon between the triangles. The visibility of the triangles is enhanced by lighting using an office lamp.

Intrinsically flat ribbons are characterized by having zero Gauss curvature and a Euclidian metric, i.e. they are developable surfaces that can be flattened without stretching and compressing. In contrast, the helicoid can not be flattened in this way, it is formed by the continuous twisting of a line as one propagates along the helicoid axis. The helicoid is also a ruled surface spanned by straight lines, but it has a negative Gauss curvature. Therefore, the ribbon and the helicoid are not isometric with each other – in the same way as a sphere is not isometric with a plane. However, the tessellations reported here allow the twisted ribbon to obtain a discrete geometry that approximately follows the helicoid surface without introducing stretching and compressions within the ribbon.

### Ribbon description

We present a few introductory remarks concerning the description and construction of all ribbons. The center curve of a ribbon will be described by a unit speed parameterization 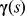, and the ribbon itself will be described as a developable surface parametrization 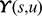 supported by the center curve in such a way that the center curve itself becomes a geodesic in the ribbon surface. The width of a ribbon is assumed to be constant and will in the following be denoted 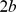.

To construct any ribbon – and hence its center curve – we will use two continuous functions, 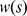 and 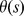 defined in the interval 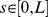, where 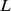 is the intrinsic length of the ribbon under construction. Throughout we assume that 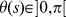 for all 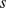 so that in particular 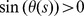 for all 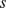. In our construction a key role is played by a unit vector field 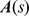, which is a field tangent to the ribbon and defined by having the angle 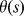 to the center curve of the ribbon. In fact, 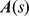 will be the direction field for the Darboux vector 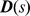 with the generating function 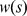 as a multiplying factor, i.e. 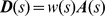.

We let 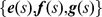 denote the unique orthonormal triple of vector solutions to the following differential system:
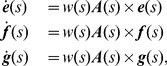
(1)where the unit vector field 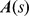 is defined in terms of 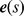 and 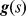 as follows:
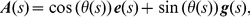
(2)and where – for uniqueness purposes – we also apply the following arbitrary initial conditions referring to a given fixed coordinate system and basis in 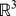:



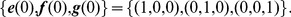
(3)The system (1) can be written explicitly as follows:
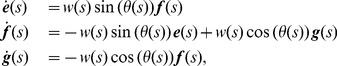
(4)where the dot notation means differentiation with respect to the unit speed parameter 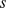. In compact matrix notation:
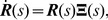
(5)where 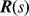 is the orthogonal matrix (with 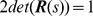) whose columns are the coordinate functions of 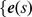, 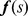, and 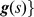 respectively, and where



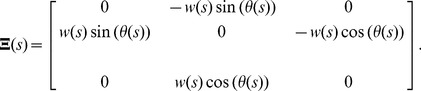
(6)The corresponding ribbon 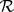 of width 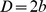 and length 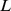 can be constructed as follows:
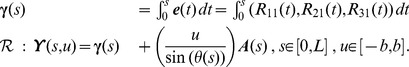
(7)


**Figure 3:**
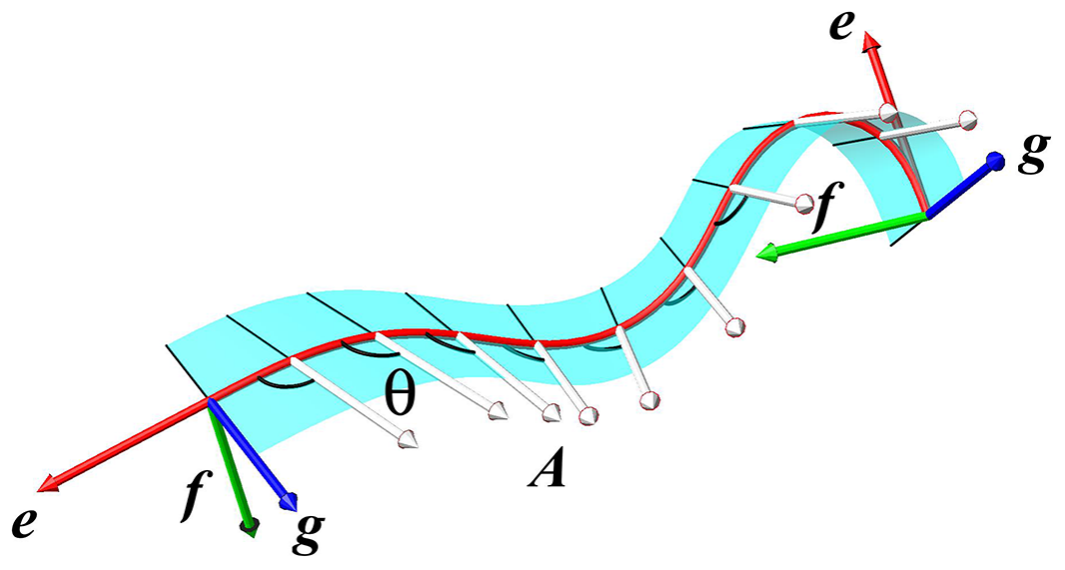
Ribbon frame. Standard setting for general ribbons with ribbon data 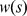, 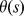. On display: 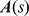, 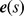, 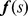, 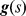, and 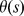.



We will refer to the frame 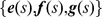 as the *ribbon frame* of the ribbon 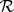. Figure 3 depicts a section of the ribbon with the 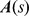 vector shown together with 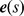, 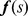, and 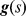. The Jacobian deformation function 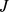 of the parametrization 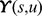 is non-singular where the ribbon surface is regular (see e.g. ref. [15]); Moreover, later we shall also need this function in order to integrate the Willmore energy along the ribbon, see equation (19).

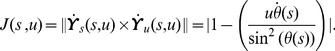
(8) Hence, in order for the ribbon to be a regular surface for 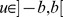 we must impose the following condition on the instantaneous direction of the field 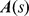, i.e. 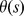, the derivative 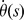, and the half-width of the ribbon 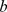:



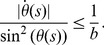
(9) The intrinsic metric tensor of the ribbon is – with respect to the 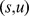 induced ribbon basis 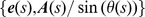:
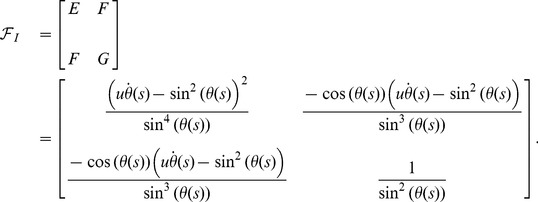
(10)


And, the matrix of the second fundamental form is:
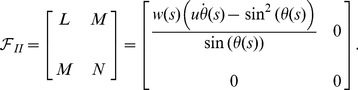
(11)


The corresponding Weingarten matrix becomes:
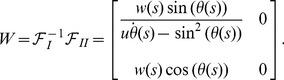
(12)


Therefore the Gaussian curvature, 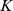, and mean curvature, 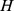, of 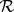 are, respectively:
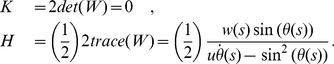
(13)


The two principal curvatures 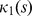, 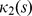 and corresponding principal directions 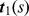, 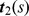 of the center curve of the ribbon are then:
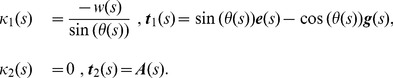
(14)


The normal curvature of the center line is, according to Euler's theorem:
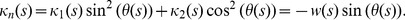
(15)


The curvature and the torsion (where 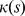 is defined to be positive, or zero, everywhere) of the center curve (considered as a space curve), are respectively:
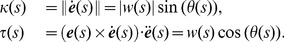
(16) Since 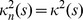 the geodesic curvature, 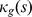, of the center curve of the ribbon vanishes:



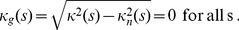
(17) The surface 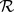 is clearly a ruled surface by construction. It is developable since the Gauss curvature vanishes identically. Hence, as the geodesic curvature of the center curve vanishes everywhere, and as we assume its half-width to be constant, 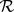 is a ribbon. The vector fields 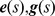 are parallelly transported (in the intrinsic metric) along the center curve.

### The ribbon frame versus the Frenet–Serret frame

When the ribbon has 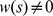 the curvature 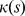 of the center curve is positive, so there exists a unique Frenet–Serret frame 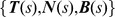 and a unique Darboux vector 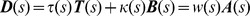 which is the instantaneous rotation vector for the Frenet–Serret frame along the center curve.

We always have 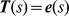 and in terms of the ribbon frame and the ribbon data we have the following correspondence with the other parts of the Frenet–Serret frame. We assume first that 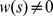 for all 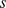 in some subinterval 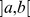 of 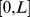. There are only two possibilities: If 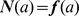 then 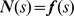, 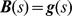, and 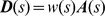 for all 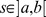. If 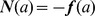 then 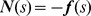, 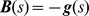, and again 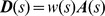 for all 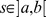. For a given ribbon the two frames can thus be identified across points with 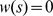 via such shifts of signs from one “side” of the 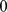-set to the other.

All the curves which can be “dressed” with a ribbon will be called ribbon curves. The set of 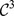 curves for which the second and the third derivatives are not simultaneously zero is one large class of ribbon curves. The differential rotations of the moving frame for the Frenet–Serret Frame, the Ribbon Frame, and the sister frame to the Frenet–Serret Frame are all three identical. I.e. they have the same vector representation, the Darboux Vector 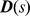. The Frenet–Serret frame is not defined at points where the curvature is zero and unfortunately it can not always be differentially patched together – some points are frame switching points. These shortcomings have been discussed in the concept of statistical models with suggestions to use a material frame [13], [16], [17] and the Darboux frame of the center line [18]. An early study involving the counting of the frame switching points gave rise to the Randrup-Røgen condition [19]. The ribbon frame discussed in this paper is obtained using two generating functions, 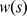, and 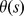 and is shown to be well behaved. Where the Frenet-Serret frame is defined we have the following relationship between the two frames. Locally they are identical except with the possibility of an inverted sign for 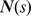 and 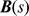. The frame switching points occur where 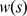 changes sign. Moreover, 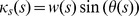 provides a convenient way to define a signed curvature for the ribbon center curve, which is a natural extension of the signed curvature conventionally assigned to planar curves.

### Short flat ribbons

Flat knotted ribbons have been studied for the Möbius band [20], the trefoil knot [21], the figure eight knot [21], and for general torus knots [22], with the purpose of finding the shortest ribbon length that can form these knot structures as flat ribbons for a given ribbon width. One way to look at such short ribbons is in the perspective of Kirchhoff-Love isotropic shells. Ribbons that can not be be shortened shall remain stationary under strain and thus support torque and forces necessary for the static equilibrium. Isotropic ribbons without any bending rigidity must therefore be either locally flat, or locally have sharp turns where our generating function 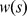 takes the value of a 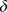-function. Plane knots are folded at these latter points while for 3-dimensional structures two tangent planes can meet along a bend. Summarizing this means that such ribbons must contain flat sections connected by bends, or folds, which resemble the observed structures in figures 1 and 2.

A similar line of reasoning supports and motivates our modeling assumptions in the next section. For a shortest ribbon we can assume that Eq. (9) must hold in the limit where an equality sign is obtained, i.e.:
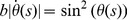
(18)


If this were not the case then a wider ribbon than the current one would have the same length. This can not be true for a twisted ribbon of minimal length.

A different perspective, though with a similar result, is obtained by studying the total Willmore energy [2]:
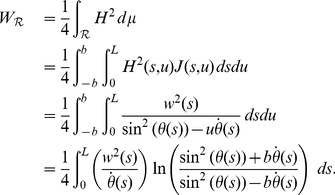
(19)


Now consider the short twisted ribbons, i.e. those for which the width can not be increased without modifying the center line, then it follows that for the stretches in 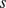 where Eq. (19) holds one can conclude that 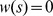 wherever 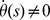 in order to prevent a divergence of 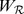. Hence, for short twisted ribbons we will assume in the following section that 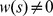 only for intervals of 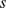 where 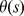 is constant, so that all the bending of the ribbon takes place as cylindrical bends. Interestingly, the assumption that a twisted ribbon is short enforces a discretization of its geometry!

### Ribbon crystals

We now discuss the observed ribbons. First their half-width parameter 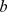 determines the scale of their structure. I.e. a ribbon scaled down by a factor of two would have half the length and half the width, but could absorb the same amount of winding for the same relative shortening. This means that two ribbons of different width but of the same length would shorten differently when twisted: The wider ribbon would shorten the most. The governing equations described above are independent of the half-width, 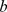, except for the inequality, Eq. (9). We can therefore conclude that the maximum length occurs when at least for part of the ribbon the limiting condition, which corresponds to an equal sign in the inequality, is valid as discussed above. We will model the limiting structure which corresponds to an upper limit for the extrinsic length as consisting of consecutive flat triangular sections merged at the edges to a full length ribbon. As will be shown below, the ideal limiting structure consists of an infinite number of triangles, which are not in accordance with observations in Figures 1 and 2. One possibility to more realistically model the material property of the ribbon is to introduce an elastic energy and minimize with respect to it [2], [23]. We choose a different strategy, namely, to introduce material properties through a geometrical parameter. This will allow us to study the phenomenon in the limit of short ribbons.

**Figure 4:**
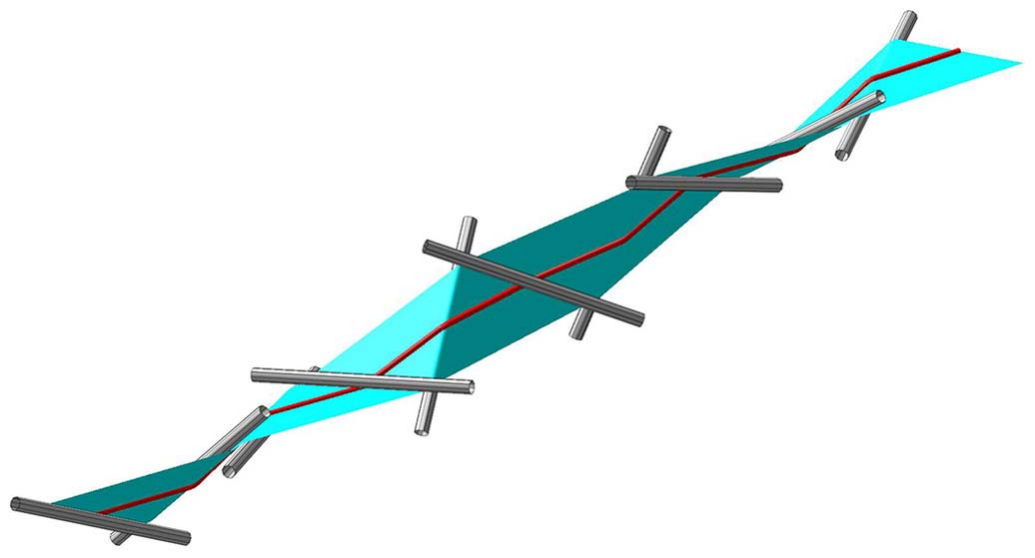
Schematic tessellation. Schematic computer drawing of a twisted triangular tessellation including the bending around thin cylindrical tubes.

In the following, it is assumed that the radius of curvature is restricted by a lower bound, 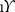, and we will study the optimum ribbon crystals as a function of 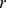. This type of assumption is a natural choice for ribbons of finite thickness, 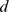, since such a condition conserves the local ribbon volume. For many materials this is a fair limit to consider, it restricts the main principal curvature accordingly 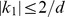. The maximally curved surfaces we can consider is then cylinder surfaces with radius 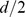. We now model the ribbon as formed by planar triangles joined together by cylinder surfaces and proceed to optimize their extrinsic length. For a typical ribbon 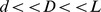, where 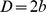 and 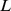 are the ribbon width and the (intrinsic) length, respectively. Figure 4 shows a schematic drawing with planar triangles and sections of cylindrical ribbons. The ribbon is repeatedly constructed of planar triangles with edges running across the ribbon. These edges make an angle 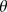 with the centerline of the ribbon. The triangles are then patched together with a thin circular cylinder surface of radius 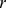 rotating around the cylinder through the angle 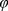. The value 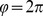 corresponds to one complete rotation around the cylinder – the orientation of the angle is given by a vector along the cylinder axis. The procedure is repeated again and again. There is a screw symmetry and the screw axis is easily found using the first two triangles. The resulting extrinsic length, 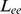, can hence be calculated and compared with the intrinsic length of the ribbon and with the total winding of the ribbon.

The ribbons that maximize 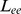 are symmetric under a discrete screw translation as well as under discrete 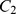 rotations. The base median of each of the isosceles triangles is a symmetry axis upon the operation of a 180 rotation. The tessellation structure is subject to a screw symmetry by which operation every isosceles triangle is mapped upon the next triangle. By two successive screw operations every triangle is mapped upon the next nearest neighbor, and then the ribbon front and back faces are also conserved. If one rotate the ribbon around one of the base medians of the isosceles triangles by 180° then the ribbon and its screw axis is maintained. Therefore, the screw axis is intersecting the base medians and is perpendicular to these. In this sense the discrete set of base medians mimic the continuous ruling of the smooth helicoid. Both the ribbon and the helicoid are described by the same pitch.

### Discrete ribbons

In the following the short ribbons for a given value of 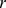 are constructed. From above, it follows that 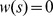 wherever 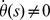 and hence we may apply 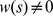 wherever 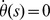 and that this latter condition holds for 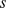 in a number 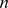 of subintervals 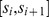 of 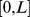, 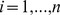. We now briefly describe the construction of ribbons with constant data. In the subintervals 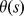 is a constant 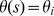 and 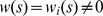 for 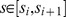, and we assume that the two sequences 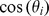 and 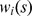 both are chosen with alternating sign so that the respective torsion-contributions 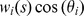 from these intervals are all positive, and that the absolute non-zero values are 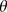 and 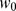.

The ribbon is built as 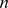 consecutive geometric units each consisting of a flat isosceles triangle with straight line center curve of length 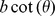 followed by a right handed bending around a cylinder of radius 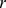, the bending having angle 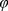, resulting in the extra contribution to the center curve length: 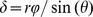. The total intrinsic length of the center curve of this construction consisting of 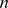 such units is then 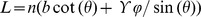.

Every geometric unit for the ribbon constructed in the above way is mapped into the next unit by a screw translation whose axis is determined as follows: Suppose the center curve of the first flat triangle in the first unit is in the direction 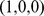 and that the triangle itself has unit normal vector 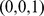. Then the axis vector of the ribbon construction is determined by the following vector:
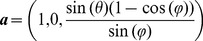
(20)


The projection of the center curve of one unit (the first, say) onto the axis has the length 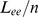, so that the expression of the total extrinsic length is easily obtained from there, see equation (21), and can be directly compared with the total intrinsic length 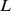 of the ribbon. For simplicity we give the expression for 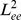:
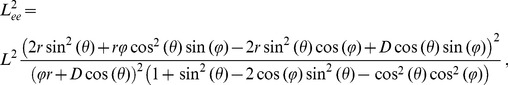
(21)where 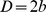 is the width of the ribbon, where the angle of rotation around the cylinder surfaces, 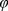, is given by



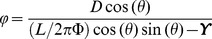
(22)and where 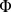 is the winding of one end of the ribbon relative to the other (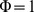 corresponds to one full turn). The winding is giving as the sum from the torsional contributions from the helical turns around the cylinders. Each one of these turns contributes an equal amount of 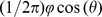.

**Figure 5:**
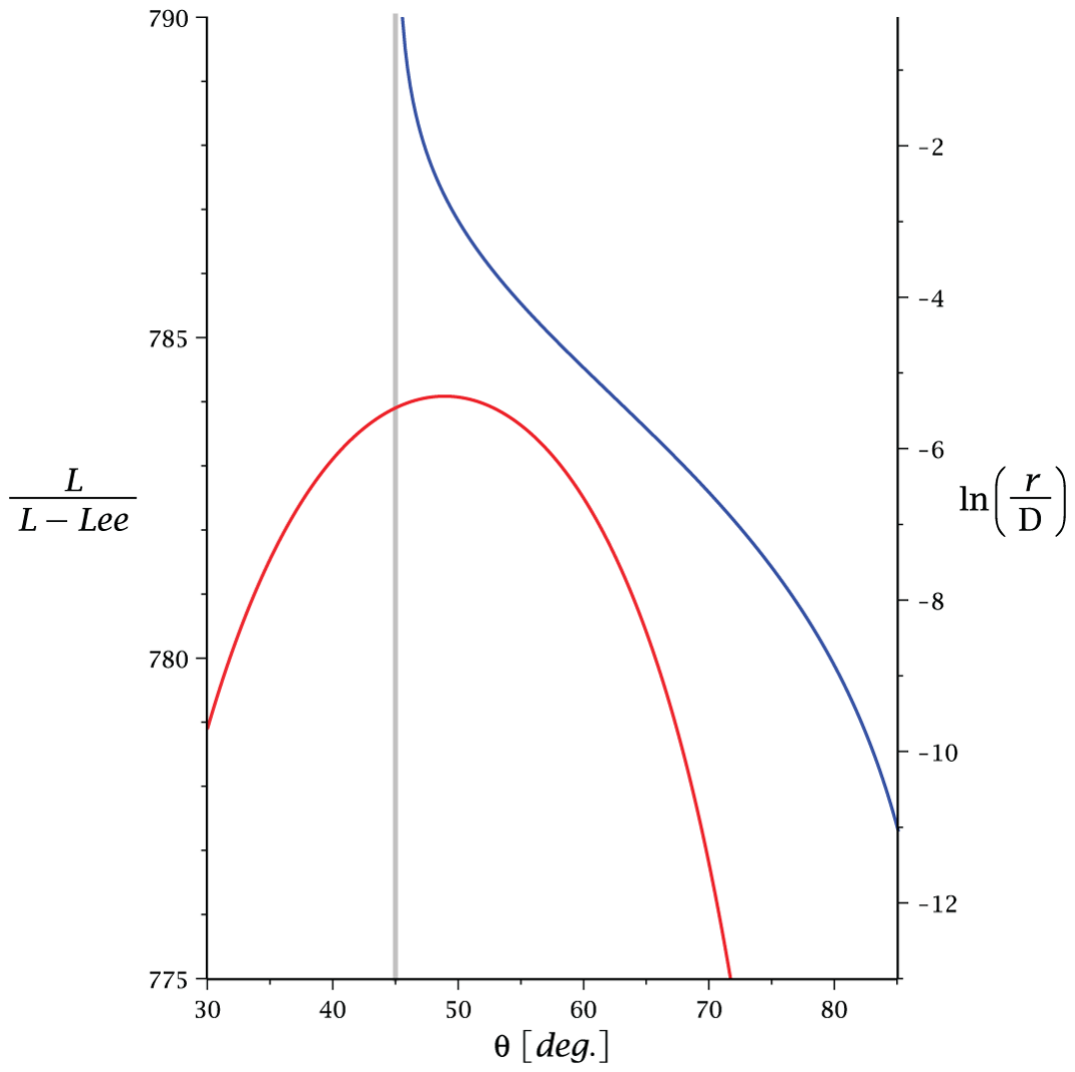
Optimal ribbon length. Left vertical axis: the red curve shows 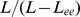 versus 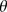, i.e. the inverse of the shortening of the extrinsic length compared with the intrinsic length as a function of the constant base angle 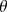 of the isosceles triangles. In the depicted calculation the cylinder radius is 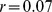, the length 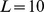, the ribbon width 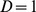, and the winding 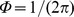. The optimum appears at the apex (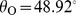) of the red curve. For smaller windings the apex moves towards 45° marked on the figure as a vertical grey line. Right vertical axis: the blue curve shows 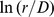 versus the optimum angle 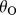. A logarithmic scale is used for the vertical axis since this is a very slow dependence. The apex of the red curve is related to the blue curve at the point 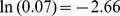 (right axis).

Figure 5 depicts 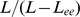 as a function of 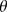 for the case 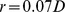, see the red curve. Notice the optimum use of ribbon at around 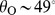. The relationship between the optimized 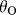 and the value of 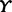 is shown with the blue curve (see right hand 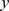-axis). We notice the very slow dependence of 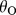 with 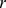 for which reason a logarithmic scale is used on the 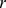-axis. For typical physical ribbons one obtains values for 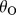 a few degrees larger than 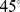. E.g. for the ribbon in Figure 2, 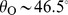. This is far from the 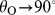 solution which corresponds to the limit 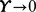. This particular short ribbon has an infinite zig-zag structure. The reason that even a minute finite 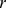 can make a significant difference to the value of 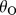, over simply using 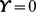, is that the contribution to 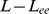 per unit of the tessellation is itself small. If one considers elastic effects then the cylinder with 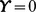 will be suppressed by the Willmore energy:
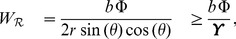
(23)where the last inequality is obtained for 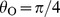 for any given value of the cylinder radius 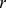. We note that the infinite zig-zag structure discussed above has an infinite Willmore energy and will therefore not appear in an elastic material.

## Conclusion

The tessellation phenomenon can be explained to result from a geometrical optimization of the end-to-end (extrinsic) length, 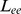.

The tessellation phenomenon can be observed with different materials including paper. One needs to use materials that are characterized by being good model systems for isometric ribbons, i.e. they bend more easily than they stretch. The observed tessellations are invariant under scaling and may be relevant for ribbons on the micro, nano, or molecular scale such as cholesterol helical ribbons [24], [25], graphene ribbons [26], and topological crystals [27]. Edge modifications of graphene ribbons [28] have been suggested to modify their twisting, and models of kinked graphene have been studied for possible new properties [29], [30]. Twisting graphene under tensile stress, as we describe above, can be an alternative way to modify its properties. Considering the about 3 Å thickness of a graphene sheet it seems interesting to work with graphene ribbons with a width of the order of 30 Å, or more. The stability of non-isometric deformations of helical ribbons has previously been subject to studies of various elastic descriptions [31]–[33]. A different example of a one-dimensional repetitive discrete structure is found in the cylindrical foam [34].

Recently, the problem of wrapping a sphere with a sheet was revisited [35], [36]. For this sheet-on-sphere challenge there is an incompatibility of the two metrics, which is also the case for the ribbon-on-helicoid system discussed here. Further, helicoids are subject to a continuous screw symmetry, the triangular tessellation described above are instead subject to a discrete screw symmetry. Interestingly, high symmetry solutions appear in several instances for the minimal ribbon length solutions. I.e. for the the non-twisted ribbon the minimal solution is a planar and straight ribbon with continuous translational symmetry. For the ribbon crystals discussed in this paper the minimal solution is invariant under a discrete screw translation. Further, for the flat torus ribbon knot [22] the minimal solution can be shown to have the symmetry of a discrete screw translation. Ribbon theory may be applicable in DNA and protein folding studies [37], [38]. Perhaps in some way not elucidated yet the relative high degree of symmetry sometimes seen in protein structures is connected with the phenomenon of being short.

It is worthwhile to comment on the long-range order of the tessellation. For the ribbon, all it takes for the tessellation to appear is a finite but arbitrary small amount of winding, 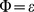. In the limit 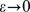 the base angles of the isosceles triangles, 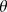, approach 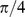. Therefore, upon an infinitesimal twisting of the ribbon the appearing tessellation has long-range order with the fundamental wave vector 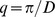. The twisted ribbon is one example which shows that an attempt to match a Euclidian geometry with a non-Euclidian geometry can generate a tessellation with long-range order. In this specific case the intrinsic structure is two-dimensional and the co-dimension is one. One may well speculate whether a similar interplay of two incompatible geometries plays a rôle in the formation of order in other systems e.g. in crystallization of crystals and quasicrystals, where indeed the latter cases are known to require several additional dimensions for their proper description [39]–[41].
